# Laser microdissection pressure catapulting (LMPC): a new technique to handle single microplastic particles for number-based validation strategies

**DOI:** 10.1007/s00216-023-04611-z

**Published:** 2023-03-04

**Authors:** Lars Hildebrandt, Tristan Zimmermann, Daniel Pröfrock

**Affiliations:** Institute of Coastal Environmental Chemistry, Department Inorganic Environmental Chemistry, Helmholtz-Zentrum Hereon, Max-Planck Str. 1, 21502 Geesthacht, Germany

**Keywords:** LDIR, Py-GC-MS, Microplastic metrology, Reference materials, Quality control, Particle sorting

## Abstract

This study examines laser microdissection pressure catapulting (LMPC) as an innovative method for microplastic research. Laser pressure catapulting as part of commercially available LMPC microscopes enables the precise handling of microplastic particles without any mechanical contact. In fact, individual particles with sizes between several micrometers and several hundred micrometers can be transported over centimeter-wide distances into a collection vial. Therefore, the technology enables the exact handling of defined numbers of small microplastics (or even individual ones) with the greatest precision. Herewith, it allows the production of particle number-based spike suspensions for method validation. Proof-of-principle LMPC experiments with polyethylene and polyethylene terephthalate model particles in the size range from 20 to 63 µm and polystyrene microspheres (10 µm diameter) demonstrated precise particle handling without fragmentation. Furthermore, the ablated particles showed no evidence of chemical alteration as seen in the particles’ IR spectra acquired via laser direct infrared analysis. We propose LMPC as a promising new tool to produce future microplastic reference materials such as particle-number spiked suspensions, since LMPC circumvents the uncertainties resulting from the potentially heterogeneous behavior or inappropriate sampling from microplastic suspensions. Furthermore, LMPC could be advantageous for the generation of very accurate calibration series of spherical particles for microplastic analysis via pyrolysis–gas chromatography–mass spectrometry (down to 0.54 ng), as it omits the dissolution of bulk polymers.

**Graphical Abstract:**

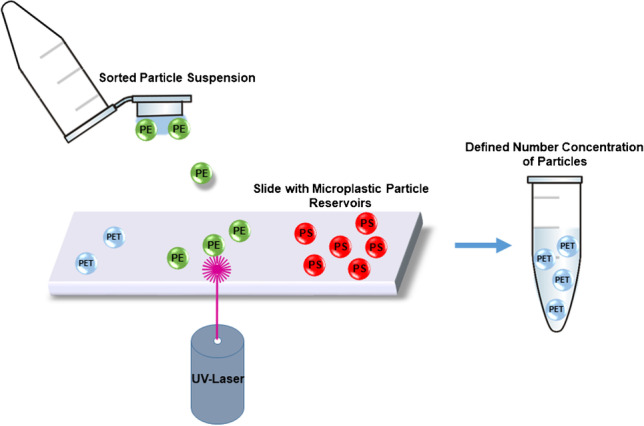

## Introduction

Reference materials (RMs) for microplastic analysis are urgently needed for the evaluation of the performance and to enable comparability of different chemical-analytical methods and sample preparation techniques [1–3]. Certification of RMs requires validated analytical methods (in terms of figures of merit, traceability, and uncertainty) for the accurate and reproducible characterization and quantification of microplastics [4]. Thus, researchers, ISO experts (e.g., ISO/TC 61/SC 14/WG 4), routine monitoring laboratories, and, last but not least, regulators face a chicken or the egg dilemma. Different matrix-matched certified RMs are required for the reliable quantification (based on number concentration and mass fractions) of environmentally relevant and realistically degraded (photooxidation and mechanical abrasion) microplastics in matrices such as water, sediments, and biota. In recent years, several research projects like EUROqCHARM (3 rounds) [5–7] and accredited RM producers such as the European Commission’s Joint Research Centre (JRC) [8] carried out important interlaboratory comparison (ILC) studies on this issue. It was shown that inhomogeneity can be a major challenge for the production of such candidate RMs. Homogeneity studies by the JRC yielded a number/mass concentration of 797 ± 151 particles L^-1^ (1 *SD*, *n* = 20) / 293 ± 41 μg L^-1^ (1 *SD*, *n* = 14) for a polyethylene terephthalate (PET) candidate RM (30–200 µm, 1 L water samples, reconstituted from a NaCl carrier) [8, 9]. This corresponds to a double relative standard deviation (2 × *RSD*) of 38% / 28% applying a level of confidence of about 95% (in accordance with ISO/IEC Guide 98-3), which is not acceptable for a RM.

A good starting point for ILCs would be candidate RMs for small microplastics (< 100 µm) whose homogeneity (*2 × RSD* between replicates) has been determined at ≤ 10% by state-of-the-art reference methods. In the past, this has not been accomplished, since often no single-particle technique but subsampling from a suspension was the method of choice. However, compared to chemicals and nanoparticles, microparticles exhibit a more heterogeneous distribution in suspension — especially without vigorous stirring.

Techniques from other research fields such as laser microdissection pressure catapulting (LMPC) could help to solve this problem. LMPC is commonly utilized in life science applications and uses two different analytical techniques commonly combined in one instrument: laser microdissection (LMD) and laser pressure catapulting (LPC). In biology and medicine, LMPC is usually applied to dissect and sort cells or parts of histological tissue preparations [10]. The technique enables cutting of the sample (placed on a microscopic slide) without heat formation in the surrounding tissue by means of a pulsed UV-A laser (wavelength 355 nm). Afterwards, the specimen is catapulted into a collection tube with a single UV-A laser shot [11]. This allows the precise handling of individual cells or particles without any mechanical contact. In fact, particles in the size range from a few to several hundred micrometers can be transported over centimeter-wide distances into a 0.25-mL centrifuge tube or another appropriate collection vial. The basic mechanism of LPC is assumed to be a gas pressure force developing under the specimen caused by the extremely high photon density within the laser spot [12].

The aim of this study was a proof of principle for the applicability of LMPC as an innovative new tool for the accurate handling and production of spike suspensions containing exact numbers of small microplastics (< 100 µm). These are urgently needed for number-based method validation in microplastic analysis. To the best of our knowledge, this work is the first application of LMPC in the context of microplastic research.

## Materials and methods

### Contamination prevention

State-of-the-art measures to mitigate contamination from the laboratory environment are described in detail in Hildebrandt et al. (2022) [13]. The protocol includes strict work in class II clean benches (Spetec GmbH, Erding, Germany; Captair GmbH, Düsseldorf, Germany), usage of pre-cleaned glass or metal laboratory equipment, air filters (DustBox 1000, Möcklinghoff Lufttechnik GmbH, Gelsenkirchen, Germany), sticky mats, lab coats, and filtration of all reagents through polycarbonate track-etched membranes (0.4 μm; Whatman GmbH, Dassel, Germany) and cellulose nitrate membranes (0.45 μm; Sartorius AG, Goettingen, Germany). Kevley slides (25 mm × 75 mm MirrIR (Low-E) microscope slides, Kevley Technologies, Chesterland, USA) were washed six times with 96% ethanol, placed in a Petri dish, and heated for 24 h at 250 °C. Type I reagent grade water (ultrapure, Milli-Q water (MQW); > 18.2 MΩ cm) was produced with a Milli-Q Integral 3 ultrapure water system equipped with a Q Pod Element and a 100 nm endfilter (Merck KGaA, Darmstadt, Germany).

### Model microplastics

Irregularly shaped high-density polyethylene (HDPE; 20–63 µm) and PET (20–63 µm) fragments (both Goodfellow GmbH, Hamburg, Germany), which were applied as in-house QC standards in recent studies [13–15], and polystyrene (PS) microspheres *d*_certified_ = 9.93 µm, *ρ* = 1.05 g mL^−1^) (Sigma-Aldrich, St. Louis, MO, USA) were used for the experiments. Physical properties, sieving and chemical characterization (elemental analysis via inductively coupled plasma mass spectrometry (ICP-MS), static light scattering analysis, visual, confocal and scanning electron microscopy (SEM), attenuated total reflection Fourier-transform infrared spectroscopy (ATR-FTIR), and laser direct infrared (LDIR) analysis of the PE and PET particles are described in previous publications [14, 16]. Highly concentrated stock suspensions (1% (*m*/*m*) (PE and PET) and 10% (PS) (*m*/*m*)) were subjected to serial dilution to achieve single microplastic suspensions containing between ~ 1300 (PE) and ~ 22000 (PS) particles mL^−1^. The concentrations were estimated by automated LDIR imaging [13, 17].

### Laser microdissection pressure catapulting

A PALM^®^ MicroBeam (P.A.L.M. Microlaser Technologies GmbH, subsidiary company of Carl Zeiss MicroImaging GmbH, Bernried, Germany) was used for the experiments. The system enables both microdissection and pressure catapulting. The applied instrument has successfully been used to isolate sensitive cell types, such as nematocysts from marine jelly fish [18, 19]. These studies showed that the laser energy (LE) and ultraviolet (UV) focus must be adjusted to the specific demands of the sample. To determine the optimal instrument settings, LE values of 60%, 70%, 80%, 90%, and 100% and three different UV laser foci were tested. For every combination, 10 particles per microplastic type were directly targeted and catapulted without being collected. Additionally, conventional microscope slides (Paul Marienfeld GmbH, Lauda-Königshofen, Germany) and specific slides (PALM^®^ MembraneSlide) covered with a UV light-absorbing membrane (polyethylene naphthalate (PEN)) were compared in the same manner.

**Fig. 1:**
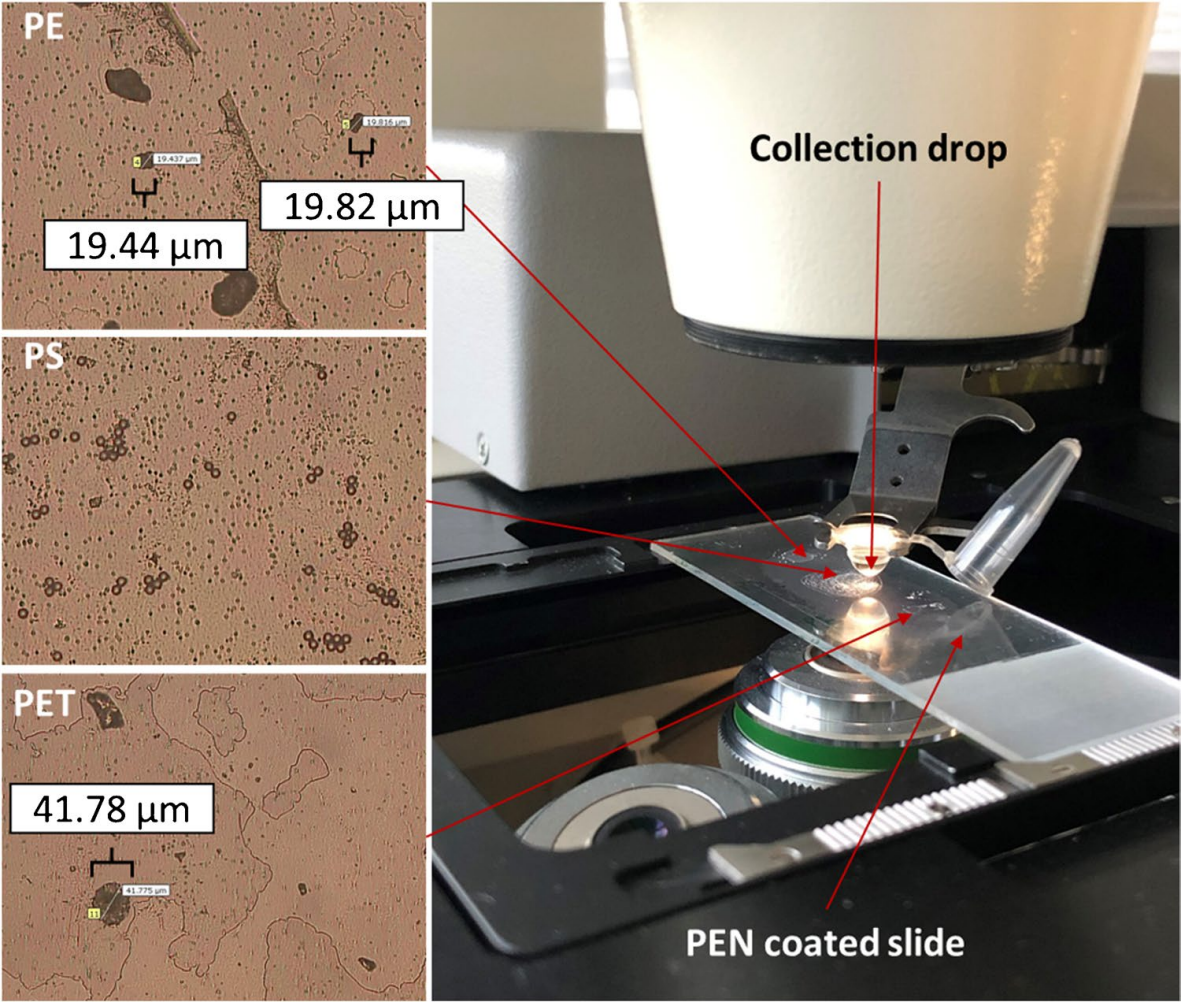
Experimental setup of the LMPC instrument (right side) used to catapult PE and PET particles and PS microspheres (left side) into a MQW collection drop. Microscope images were taken at 20-fold magnification

The optimized settings (LE = 100% and UV focus: 70) were applied to catapult six replicates of 1 particle (6 × 1 each) and three replicates of 2 (3 × 2 each) particles of PE and PET (Fig. 1) into a MQW drop of ~ 25 µL (one drop per replicate) placed at the inner side of the tube lid (0.25 mL Eppendorf, Hamburg, Germany). Moreover, 1, 2, 3, 4, 5, 6, 7, 8, 9, and 10 PS microspheres were sequentially catapulted into 10 different drops. The drops were inspected at 5-fold magnification to see the fast-moving particles (Brownian motion). Subsequently, the drops were transferred onto a Kevley slide. No rinsing was conducted since moderate contact between the drop and the slide was sufficient to place the entire collection drop on the slide. To generate a blank and exclude potential artifacts or interferences from the PEN membrane, the membrane was shot 3 × 10 times without targeting any particles.

### LDIR imaging

LDIR imaging was applied to chemically identify the microplastics. Furthermore, LDIR was applied for size determination and counting of the particles after pressure catapulting. The operation principles of LDIR are described in previous publications [13, 17, 20, 21]. The slides were scanned at the wavenumber 1799 cm^−1^. Particles were manually localized based upon the IR contrast. Three spectra were acquired per particle and compared to the spectral database (hit quality value: comparison of 1st derivatives, Pearson correlation coefficient). The Hereon LDIR Library for Microplastic Analysis [22] was used to determine the polymer types after LMPC. The library includes various spectra of the in-house QC standards. Additionally, an automated analysis (particle size range 10–500 µm) of entire slides was carried out to check if any artifacts from the PEN membrane or particle fragmentation occurred.

## Results and discussion

### Method optimization

To find the optimal instrument settings, various LE values and UV laser foci were tested. A LE of 100% turned out to be optimal for the LMPC process of PE and PET microplastics. 65% LE was sufficient for ablating small PS spheres (10 µm) but not for the larger PE and PET fragments with sizes between 41 and 65 µm as shown in Table 1. At LEs < 100%, these particles were often not ablated from the slide or did not move at all. To set the focus of the UV laser below, the microplastics was also crucial. A UV focus of 70 was optimal for the studied particle sizes and polymer types.

**Table 1:** Size and spectroscopic analysis of six quantitatively ablated PET and PE particles. Two particles each were ablated with one laser shot for subsequent analysis via the LMPC (left side) and the LDIR (right side) with regard to their sizes and IR spectra

Instrument	LMPC		LDIR		
Particle	Shortest dimension [µm]	Longest dimension [µm]	Shortest dimension [µm]	Longest dimension [µm]	Hit qualityvalue (0–1)
PET particles					
*1*	60	93	96	105	0.94
*2*	57	88	73	89	0.95
*3*	58	65	67	81	0.95
*4*	49	51	73	74	0.93
*5*	63	85	57	101	0.95
*6*	60	81	72	109	0.92
*Mean*	58	77	73	93	0.94
PE particles					
*7*	48	56	81	88	0.98
*8*	51	60	47	71	0.98
*9*	41	55	58	58	0.98
*10*	41	70	62	63	0.99
*11*	45	46	53	59	0.98
*12*	37	48	55	58	0.98
*Mean*	44	56	59	66	0.98

**Fig. 2:**
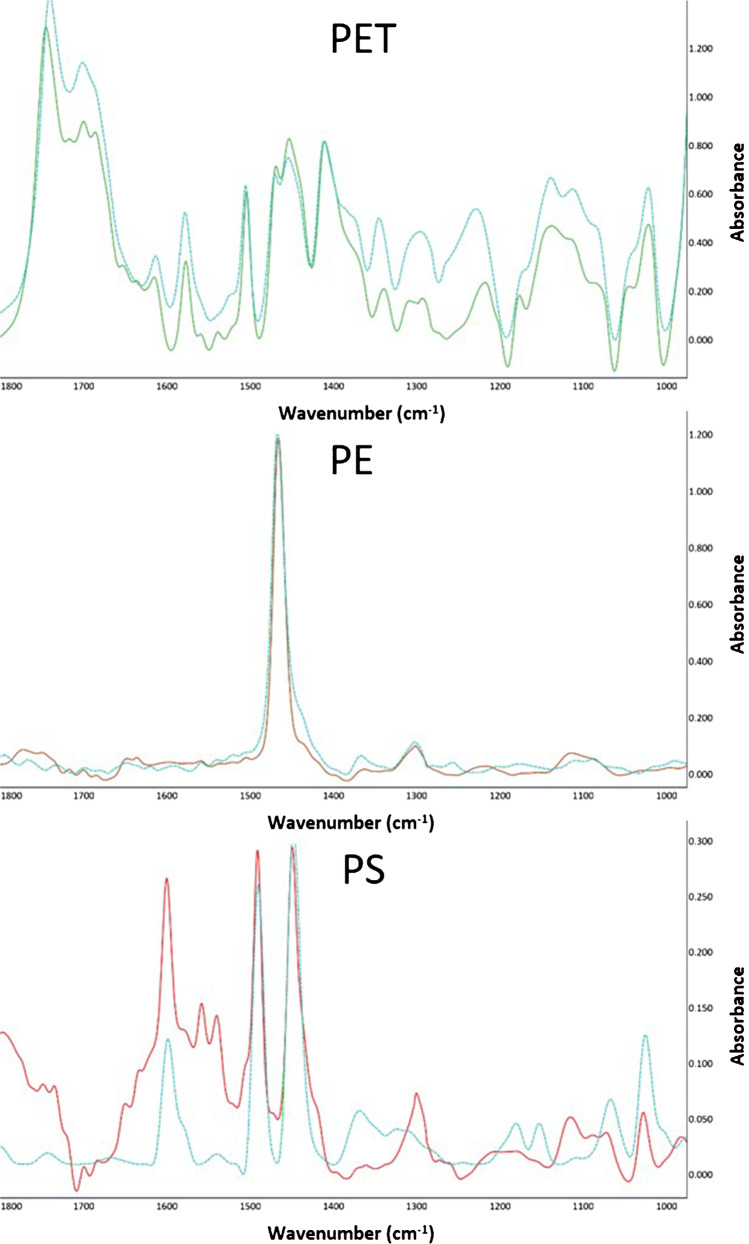
Acquired LDIR spectra (solid lines) and best-matching reference spectra (dashed lines) of single microplastic particles of the used polymer types (PET, PE, and PS) after their transfer via LPC

Additionally, working with PEN-coated slides was necessary to prevent unwanted particle fragmentation. For LMPC of particles deposited on a conventional microscope slide, strong particle destruction and fragmentation were observed at 20-fold and 40-fold magnification, respectively. However, the PEN coating (strong UV absorption) enabled a mild LMPC without fragmentation (particle shapes remained the same). Furthermore, the ablated particles were analyzed via LDIR for possible changes in the particles’ IR spectra, as shown in Fig. 2. Based on these spectra, no chemical alteration of the ablated particles was evident. The fact that LMPC even enables isolation and enrichment of intact cells or organelles [11, 23] underpins its mild ablation process. Nonetheless, minor changes with respect to oxidation state, charge, hydrophobicity, and the fine structure of the microplastics’ surface cannot be excluded.

## Recovery experiments with polyethylene and polyethylene terephthalate fragments

**Fig. 3:**
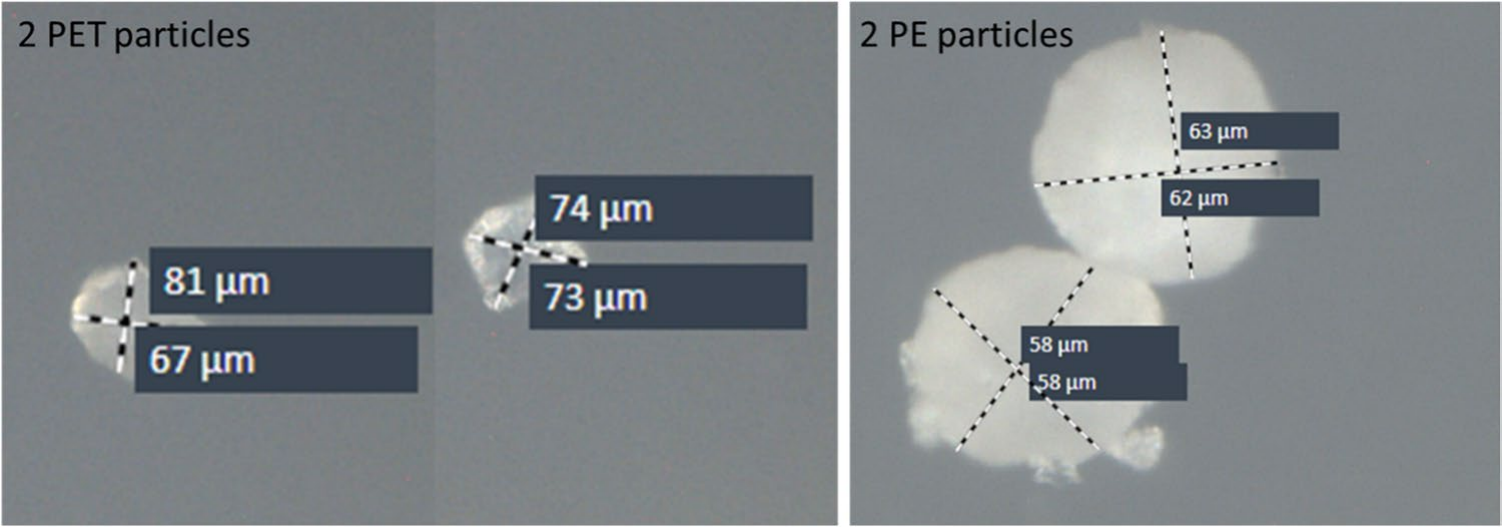
Visual image of two PET (left) and PE particles (right) acquired by the LDIR including particles sizes

This study provides the proof of principle that LMPC is capable of catapulting defined numbers of microplastic particles into a collection drop. All particles that were visually confirmed to be in the collection drop (by means of the LMPC’s microscope) could be transferred onto a Kevley slide and were chemically identified by LDIR imaging (in the automated and manual mode). After the catapulting of a single (*n* = 6 each) and two PET and PE particles (*n* = 3 each), all microplastics were recovered on the Kevley slides. The particles were unambiguously identified by LDIR analysis with hit quality values (spectral matching with the library spectrum) ≥ 92% (Table 1 and Fig. 2). High-magnification images were also recorded using the microscope-grade objective (Fig. 3).

**Fig. 4:**
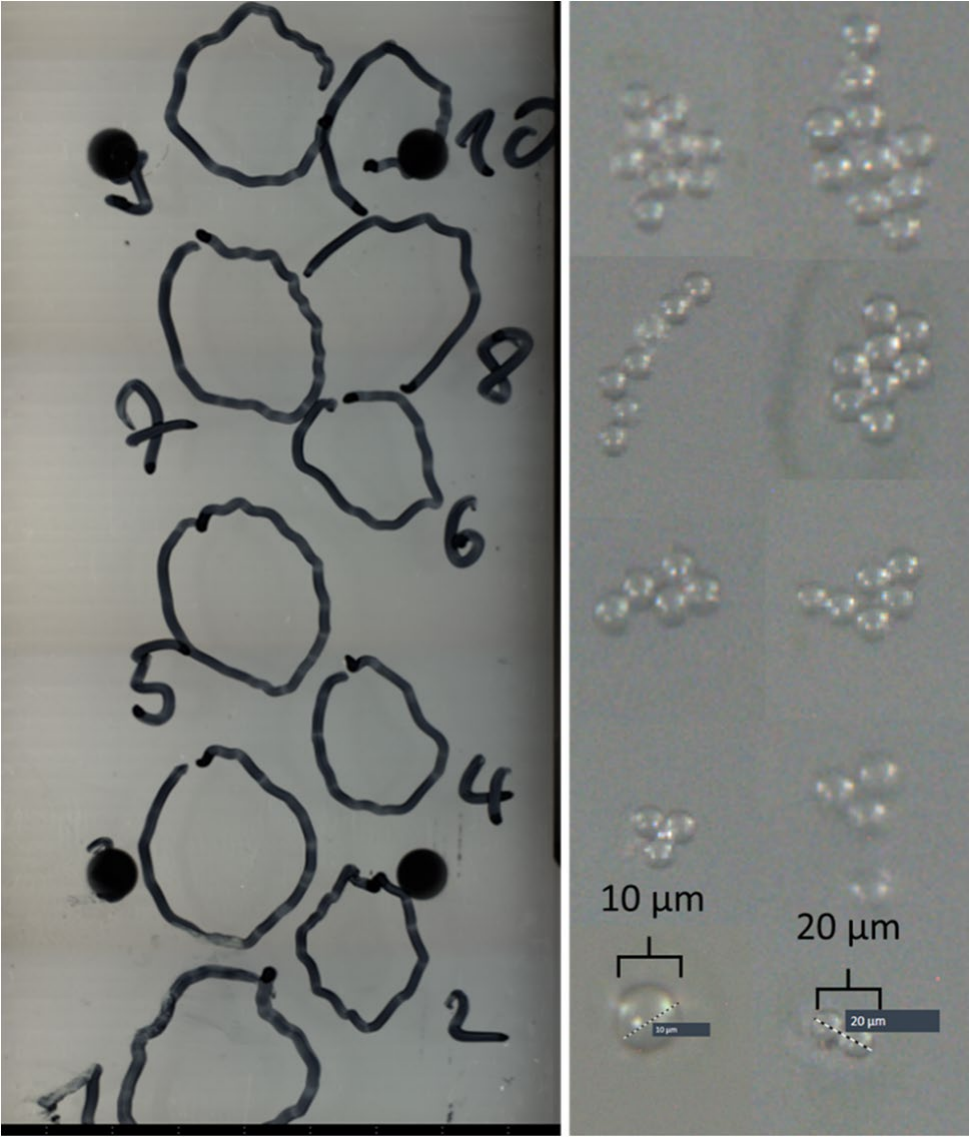
Kevley slide (left) containing 1–10 PS microspheres that were catapulted into a collection drop (numbers represent the quantity of PS microspheres). All aggregates were quantitatively catapulted by one single laser shot each. LDIR images of the ablated PS aggregates are shown right

No fragmentation and no artifacts from the PEN membrane were observed when the slides were analyzed by automated LDIR imaging (size range 10–300 µm). Only the catapulted microplastics (100% recovery) and the permanent marker lines (example shown in Fig. 4) were detected. Due to reports on underestimation of particles < 60 µm [20], additional IR spectra were acquired manually for every IR contrast pattern (resolution: 1 µm; scan wavenumber 1799 cm^−1^). This workflow is time-consuming but enables quantitative detection of particles down to 10 µm.

Interestingly, systematic size differences were observed between the determination using the LMPC and manual LDIR analysis (Table 1). When two particles were catapulted into a drop, the PET particles’ average shortest and longest dimensions of LMPC measurements were smaller by 15 µm and 16 µm than LDIR measurements. For the PE particles, the differences between LMPC and LDIR measurements were 15 µm and 10 µm. Nonetheless, the shapes of the particles did not change, and no small particles were observed in the IR images (resolution: 1 µm) and by means of the LMPC’s microscope (20- and 40-fold magnification). The differences probably stem from either the different contrast of the instruments, the selection of the shortest and longest dimensions, which is somewhat subjective, or the fact that the particles could only be inspected in two and not three dimensions.

As shown in Fig. 3, the PE particles tended to form loose aggregates — independent from the catapulting process. This was observed for both the collection drop and the imaged Kevley slides. The fact that LMPC also works with PE and not only with PET and PS particles demonstrates that no aromatic molecules, which show strong UV absorption, are required in the polymer backbone for a proper application of the LMPC.

## Recovery experiments with polystyrene microspheres

It was possible to accurately catapult 1, 2, 3, 4, 5, 6, 7, 8, 9, and 10 PS microspheres (10 µm diameter) into 10 different collection drops. As shown in Fig. 4, the different particle numbers were catapulted as aggregates with a single shot. The diameter of 10 µm was confirmed by LDIR measurements (Fig. 4; for catapulted particles) and the LMPC microscope (before the catapulting).

Spherical microplastic particles do not account for a high share of environmental microplastics since these are mainly of secondary nature. Nonetheless, using well-defined spherical particles could be advantageous for the generation of very accurate calibration series or spike suspensions by LMPC. The catapulted PS particle number series corresponds to a well-defined 10-point calibration with PS masses ranging from 0.54 to 5.4 ng (based on the certified data: *r* = 5.38 µm, *ρ* = 1.05 g mL^−1^). Especially in very low but environmentally realistic concentration ranges, calibration of pyrolysis–gas chromatography–mass spectrometry (Py-GC-MS) is challenging, which is why some researchers use nanoplastics for calibration [24–27]. On the one hand, nanoplastics show a more homogeneous distribution and stability in suspension. On the other hand, they might behave differently in the pyrolysis process. Furthermore, our approach could omit the dissolution of polymers, which can be quite challenging and often requires toxic solvents as well as high temperatures hampering usage of liquid standards [28]. Currently, different specialized suppliers (e.g., Cospheric LLC (CA, USA)) commercially offer microplastic standards such as PE, polypropylene, and poly(methyl methacrylate) microspheres, which could be used for LMPC-based calibration approaches.

## Extended utility of LMPC for method validation, environmental samples, and reference materials

In method validation, there is often a mismatch between the particle properties (such as concentration and particles size) of the QC suspension and the samples of interest, such as water, sediment or biota [29, 30]. LMPC enables spiking of realistic and well-defined numbers of small microplastics (< 100 µm) for recovery experiments or internal standard addition. It allows the production of spike suspensions with single or multiple polymers and known number concentrations.

Moreover, LMPC might enable sorting of individual microplastics extracted from complex environmental samples. After the analysis of a slide by Raman [31], FTIR [32], or LDIR imaging, microplastics could be enriched in the collection drop, e.g., for further chemical (e.g., additives) or biological (e.g., biofilm) analyses. Nonetheless, this would require microplastic analysis on the PEN membrane or optimization of the LMPC process for other slide types.

**Fig. 5:**
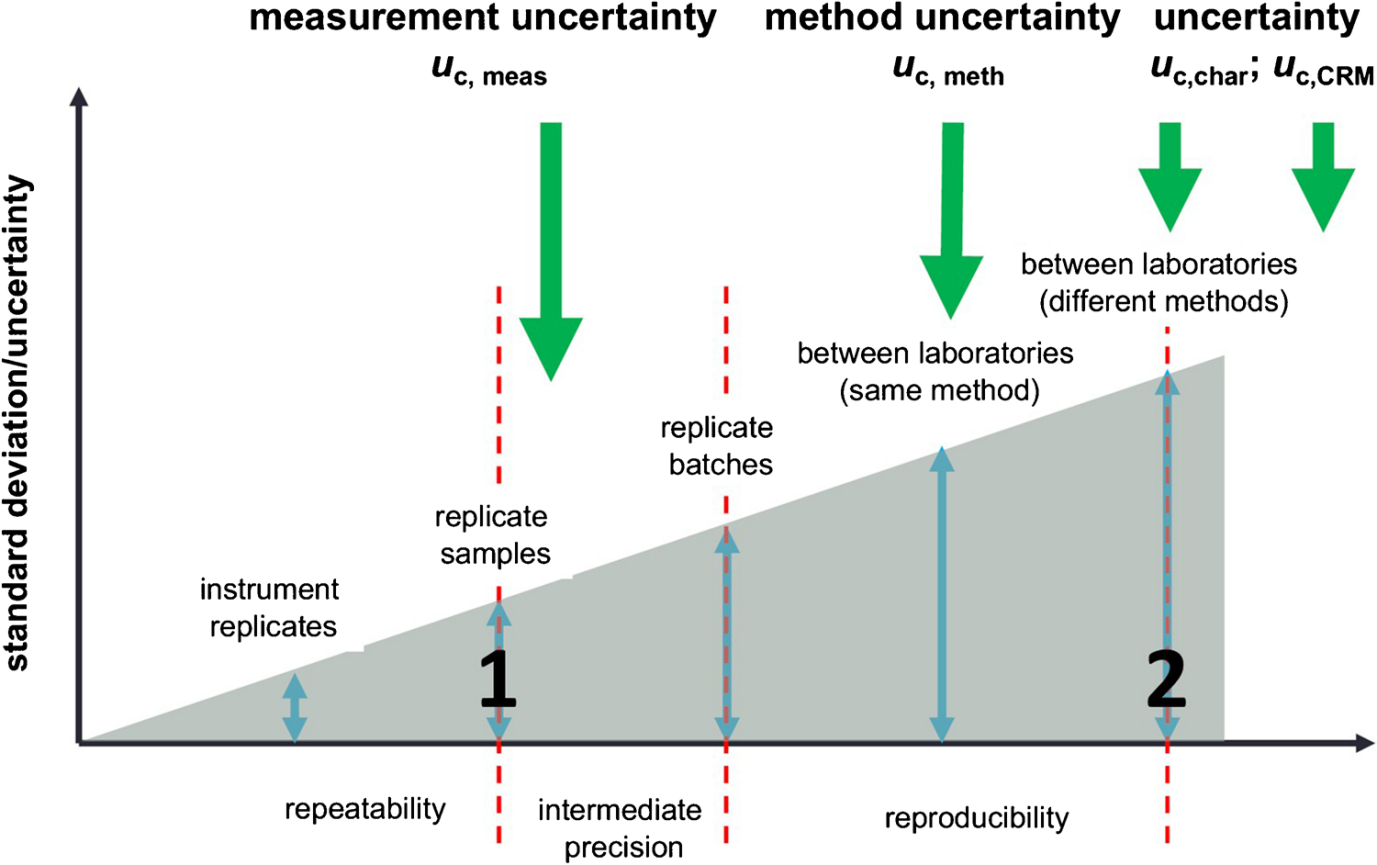
The uncertainty hierarchy. Modified from [35]

We see the highest potential of LMPC in the production of microplastic RMs, which has proved to be challenging in the past. The first concrete attempt at official monitoring of microplastics in water, the California Safe Drinking Water Act (SB 1422), demands for a “Standardized Analytical Method for Microplastics in Drinking Water” [33]. Therefore, certified microplastic RMs are mandatory to achieve global method harmonization. Different national and international metrology institutes aim for the production and certification of such RMs. Consequently, ILC studies (round robin tests) are conducted to compare different analytical methods and enable a top-down uncertainty evaluation. In the past, it has been shown that certification is hampered by large uncertainties both in the production and in the analysis of microplastic candidate RMs. In an ILC by WEPAL-QUASIMEME/NORMAN on microplastics (in soda tablets), 29–91% *RSD* regarding the particles numbers detected in the samples (soda tablets) was observed between the different laboratories (*n* = 30) (**2** in Fig. 5) [34]. It is noteworthy that the preceding homogeneity investigations (reference analysis) revealed double *RSD*s between 22 and 76% for the microplastic candidate RMs (**1** in Fig. 5).

Our data shows that this high uncertainty in the production could be greatly reduced by LMPC. The technique could circumvent the problem of potential inhomogeneity that occurs during sampling of microparticles (insufficient homogenization by stirring) from a stock suspension to bring science one step closer to a certified microplastic RM.

## Conclusion

The conducted proof-of-principle highlights the benefits of LMPC for future research related to the analysis and handling of microplastics. Experiments do not indicate any chemical or physical alteration of the studied model microplastic particles, which is a prerequisite for future in-depth studies. Future in-depth studies should clarify if LMPC may generate any unwanted small microplastic particles (e.g., by Raman imaging) or nanoplastics (e.g., by SEM) which cannot definitively be excluded based on our current data. This also applies for any potential very small artifacts stemming from the PEN membrane. Future studies should also try to achieve a workflow for conventional microscopic slides and a higher degree of automation. Currently, the process remains time-consuming since only one collector cap can be handled which hampers upscaling (approximately one minute per microplastic particle). Additionally, the long-term stability of the spike suspensions should be evaluated.

Possible applications may involve generation of urgently needed RMs, spiking of internal standards, calibration of analytical instruments like Py-GC-MS, or even the handling and sorting of environmental microplastic samples before further analysis. Routine application to environmental samples would require almost quantitative matrix removal. Thus, there are currently more obvious applications regarding analytical method validation and harmonization. We are convinced of the important future contributions of this technology in microplastic research.
